# Early infant diagnosis of HIV infection using DNA-PCR at a referral center: an 8 years retrospective analysis

**DOI:** 10.1186/s12981-016-0112-0

**Published:** 2016-09-08

**Authors:** Tolessa Olana, Tigist Bacha, Walelign Worku, Birkneh Tilahun Tadesse

**Affiliations:** 1 Addis Ababa University, Addis Ababa, Ethiopia; 2 Department of Pediatrics, Addis Ababa University, Addis Ababa, Ethiopia; 3 School of Public Health, Addis Ababa University, Addis Ababa, Ethiopia; 4 Department of Pediatrics, Hawassa University, P.O. Box: 1560, Hawassa, Ethiopia

**Keywords:** Mother to child transmission, HIV, DNA PCR, Ethiopia

## Abstract

**Background:**

Over the last decade, Ethiopia adopted different strategies of prevention of mother to child transmission of HIV (PMTCT). Prior to implementation of Option A in 2011, there was no provision of prophylaxis for PMTCT. With ‘Option A’, PMTCT interventions relied on maternal CD4 count. In early 2013, ‘‘Option B+’’ has been started; with this option, antiretroviral therapy is started and continued for life to any HIV positive pregnant mother irrespective of CD4 count with an enhanced treatment for the baby. Though there are a number of studies which evaluated the effectiveness of PMTCT interventions, the current study assessed the real-world effectiveness of PMTCT options in a setting where there is limitation of resources.

**Objective:**

This study tried to address three questions: what proportion of babies tested by DNA-PCR are HIV infected in the first 2 months of life? How does the type of PMTCT intervention affect presence of HIV infection at this age? What are the factors affecting HIV transmission, after controlling for type of PMCT-HIV intervention?

**Methods:**

We assessed records of 624 registered HIV exposed infants and 412 mothers who were delivered at Bishoftu Hospital from May 2006 to August 2014. Presence of HIV infection at 6–8 weeks of age was assessed from the records. Maternal and infant risk factors for infection at this age were analyzed. Data were collected using standard data abstraction format and were analyzed using SPSS version 20.

**Results:**

Among all the infants who were delivered at the hospital during the study period, 624/936 (66.7 %) had undergone early infant diagnosis at 6–8 weeks. Twenty-seven (4.3 %) were positive for HIV DNA PCR at the age of 6–8 weeks. None of the infants who received ‘‘Option B+’’ had a positive HIV DNA PCR result. HIV infection rate was highest among those who took either no prophylaxis or single dose Nevirapine (11.5 and 11.1 % respectively). Those who took single dose Nevirapine and Zidovudine had HIV positivity rate of 3.9 %. Many of the covariates which were shown to be predictors on bivariate analysis were found not to be independent predictors on multivariate analysis.

**Conclusion:**

PMTCT ‘’Option B+’’ resulted in zero HIV infection rates among the included infants. There was a high loss to follow up rate at 6–8 weeks of age. The authors recommend that a better strategy of linkage to care and treatment should be devised for HIV exposed infants.

## Background

In 2011, United Nations General Assembly Special Session (UNGASS) placed a clear emphasis on the effect of HIV/AIDS on maternal and child health. The final declaration of commitment from the assembly stated is to reduce the number of children newly infected with HIV by 90 % by 2015 [1]. Mother-to-child transmission (MTCT) of HIV accounts for 14 % of all new HIV infections worldwide, and may occur during pregnancy, labor and delivery or breastfeeding. In the absence of prevention, rates of MTCT are estimated to be 25–45 % [2, 3]. In 2012, according to the Ethiopian Health and Nutrition Research Institute, MTCT rates were 15 and 30 % without and with breast feeding respectively [4]. Timely initiation of PMTCT interventions dramatically improved the natural history of perinatal infections [5, 6].

Ethiopia is among countries working to achieve the goal set by UNAIDS in 2011, the Joint United Nations Program on HIV/AIDS; a global call for the elimination of MTCT by 2015 [2, 7, 8]. Over the past couple of years, Ethiopia adopted the different strategies of PMTCT. It has been implementing the 1 year accelerated PMTCT plan for ‘Option A’ since December 2011. ‘Option-A’ treatment or prophylaxis is dependent on CD4 count. It requires a variety of drugs across the continuum which creates complexity in patient management. Since early 2013, Ethiopia launched the ‘‘Option B+’’ PMTCT approach which proposes the same triple antiretroviral drugs to all HIV-infected pregnant women, beginning in the antenatal clinic setting, but also continuing this therapy for all these women for life without need for an initial CD4 test and a 6 week Nevirapine therapy for the infant [9].

While many countries still practice the WHO 2010 guidelines with either Option A or B, the PMTCT and cost effectiveness of Option B+ has been increasingly reported [10–13]. Gopalappa et al. described the cost effectiveness of Option B+ comparing it with Option A and B. It was the most cost-effective strategy costing between $6000 and $23 ,000 per infection averted compared with Option A. Option B+ averted more child infections compared with Option B and costed less than Option B. Considering adult sexual transmissions averted, Option B+ was found to cost less and averted more infections than both Options A or B [11]. Though many of the studies indicated that Option B+ is an effective PMTCT approach, its real-world performance might be different because of differences in patient socio-demographics and the availability of resources for patient follow up care.

In the current study, we hypothesize that the real-world effectiveness of Option B+ PMTCT in preventing MTCT of HIV in the first few months of life is higher than the previous approach of Option A. The current study was done to assess the real world effectiveness of Option B+ in preventing MTCT in a setting where resources are meagre and prevalence of HIV is high as compared to the developed world. Due to lack of resources for early infant diagnosis, in the Ethiopian context, many HIV exposed infants do not undergo a second DNA PCR for HIV test after the first 6–8 weeks test. We assessed the rate of positivity of the first DNA PCR for HIV done at 6–8 weeks of age based on the existing national early infant diagnosis guidelines of HIV infection; and the effectiveness of different strategies for PMTCT over the past 8 years at one of the largest hospitals in Ethiopia. We also assessed the associated factors of DNA-PCR positivity for HIV among HIV-exposed infants at Bishoftu hospital.

## Methods

Data collection was conducted at Bishoftu hospital from November to March 2014. Bishoftu city has a total population of about 128,400 in 2013 [8]. Bishoftu hospital is one of the oldest health institutions in Ethiopia; it serves as referral center for 10 health centers with a catchment of over 1 million people. Apart from other services, the hospital is providing HIV chronic care including PMTCT services since 2004. In the area, the adult prevalence rate of HIV is estimated at 2.4 % and the incidence rate is 0.29 %. The urban prevalence rate is higher and is estimated at 7.7 %, while the rural prevalence rate is 0.9 %. The prevalence rate is 1.7 % for males and 2.6 % for females. With 90,000 HIV-positive pregnant women, there are an estimated 14,000 HIV-positive births and a total of 28,000 AIDS death annually [14].

A retrospective record review was done among infants and their mothers from the HIV exposed infant registers of the Hospital. All records of HIV-exposed infants registered between May 2006 and August 2014 at Bishoftu hospital were included in the study. All records of exposed infants and their mothers were included. Excluded were those infants with no DNA PCR result at 6–8 weeks, missing infant/mother charts and those who had DNA PCR result after 8 weeks since they were very few in number.

There is a separate HIV exposed infant registration form. The registers are both paper and computer based. The PMTCT nurse fills the HIV exposed infant form which is distributed by the Ministry of Health and the data clerk enters individual patient data to a computer based database. The registration started in 2001 at three hospitals in the country [15]. From infants’ registers, mothers’ record numbers were documented and hence, allowed for retrieval of respective maternal records of each infant. The electronic medical record of the mother and the corresponding infants were cross-checked for the availability of their data and were linked with infant records in order to confirm real mother infant pair.

Information on demographic characteristics and uptake of different components of the PMTCT package were obtained from HIV exposed infant follow-up charts, HIV care/ART follow up charts and intake forms, and antenatal patient charts using data abstraction forms.

To ensure quality of data, the abstraction tool was pre tested and all were checked for completeness before data entry. Data abstraction was made by a team of trained nurse data collectors. Supervision during data collection and data entry were conducted. Quality of collected data was continuously checked by the principal investigator and random checks by one of the co-authors. Errors and consistency checking procedures for the data were controlled during analysis. Data were checked, cleaned, coded and entered in EPI info version 3.5.1 and were exported to SPSS version 20 for further analysis.

Independent variables were tested for predicting rate of positivity of DAN PCR for HIV using bivariate logistic regression. Then all variables having p ≤ 0.05 in the bivariate analysis were further entered into multivariate logistic regression model and variables which had p < 0.05 were retained as independent predictors of positivity of DNA PCR at 6–8 weeks.

In the current study, MTCT of HIV was considered as transmission of HIV from HIV positive mother to her child during pregnancy, childbirth and early breastfeeding. PMTCT intervention refers to the use of ART or ARV drugs in mother and infant to reduce MTCT of HIV. “Loss to follow up” was defined in the current study as not showing up for early infant diagnosis or other HEI care and treatment after the initial registration. A mother was considered as taking the various options based on the information given in Table 1 [16]. Ethiopia adopted Option A in 2011 and then directly changed to Option B+ in 2013. Hence, there was no experience with Option B [15].

**Table 1:** The PMTCT options and interventions for the mother and baby

	Treatment (for CD4 count <350 cells/mm^3^)	Prophylaxis (for CD4 count >350 cells/mm^3^)	Infant receives
Option A	Triple ARVs starting as soon as diagnosed, continued for life	Antepartum: AZT starting as early as 14 weeks gestation Intrapartum: at onset of labour, single-dose NVP and first dose of AZT/3TC Postpartum: daily AZT/3TC through 7 days postpartum	Daily NVP from birth until 1 week after cessation of all breastfeeding; or, if not breastfeeding or if mother is on treatment, through age 4–6 weeks
Option B	Triple ARVs starting as soon as diagnosed, continued for life	Triple ARVs starting as early as 14 weeks gestation and continued intrapartum andthrough childbirth if not breastfeeding or until 1 week after cessation of all breastfeeding	Daily NVP or AZT from birththrough age 4–6 weeks regardless of infant feeding method
Option B+	Triple ARVs starting as soon as diagnosed, continued for life	Triple ARVs starting as soon as diagnosed, continued for life	Daily NVP or AZT from birth through age 4–6 weeks regardless of infant feeding method

Since the age at first DNA PCR test was not directly recorded, it was calculated from the records using the date of birth column and the date when sample for DNA PCR testing was collected.

## Ethical consideration

Ethical clearance was obtained from Institutional Review Board (IRB) IRB of University of Gondar. Permission letter was obtained from the management Committee of the Bishoftu Hospital (Ref.BH-219/2007 issued 15/5/2007) prior to data collection. Since the study utilizes routinely collected, aggregated program data at the hospital, confidentiality of patient information was ensured as the names or identification number of study participants was not included in the data collection format.

## Results

A total of 936 HIV exposed infants were registered at Bishoftu hospital during the study period. Among these, records of 624 exposed infants and 412 mothers were included for the study. One hundred ninety-nine (22.2 %) were lost to follow up care and treatment; the rest 113/936 (12.1 %) were excluded because they didn’t have DNA-PCR result for HIV at the age of 6–8 weeks or had incomplete records.

Among the infants included in the analysis, 323/624 (51.8 %) were male. Included infants were 6 weeks, 574/624 (92 %); 7 weeks, 8/624 (1.3 %); and 8 weeks, 42/624 (6.7 %) of age at the time of DNA PCR for HIV test. The majority, 295 (47.5 %) of the mothers were on antiretroviral therapy during pregnancy while 50/624 (8 %) were on single dose Zidovudine for PMTCT purpose. Mothers who have not received any PMTCT ARVs were 123/624 (14.9 %) during ANC and 95/624 (15.2 %) during labor. Most of the babies, 525/624 (84.1 %) were delivered at health institutions.

The majority, 494/624 (79.2 %) were on exclusive breast feeding whereas, 91/624 (14.6 %) and 29/624 (4.7 %) were on exclusive replacement and mixed feeding respectively. Based on the country’s guidelines at the time of enrolment, 305/624 (48.9 %) of the infants were on the former WHO recommendation of single dose Nevirapine or single dose Nevirapine with Zidovudine. The remaining 179/624 (28.7) and 12/624 (1.8 %) were on Nevirapine for 6 weeks and Nevirapine for greater than 6 weeks respectively. No prophylaxis was given to 90 (14.4 %) infants of which 42/90 (46.67 %) were born at home. Unfortunately, 48/90 (53.33 %) of those infants who didn’t receive any prophylaxis were born at health facility (Table 2). The mean CD4 counts of mothers of babies who were positive for HIV DNA-PCR and those who were not at 6–8 weeks were comparable (415.00 (SD = 331.47)/mm^3^ and 418 (SD = 239.09); p > 0.05 respectively). All HIV exposed infants were initiated on Co-trimoxazole at enrollment.

**Table 2:** Characteristics of mothers and infants included in the study during the specified study period (2006–2014) at the PMTCT/ART center of Bishoftu Hospital

Variable	Frequency (N)	Percentage (%)
*Sex*		
Male	323	51.8
Female	301	48.2
*Place of birth*		
Health facility	525	84.1
Home	55	8.8
Unknown	44	7.1
*Type of infant feeding*		
Exclusive breast feeding	494	79.2
Exclusive complementary feeding	91	14.6
Mixed feeding	29	4.7
Weaned of breast feeding	2	0.3
*Mothers CD4 during ANC* ^a^		
Less than 100	15	2.4
101–300	73	11.7
301–500	124	19.9
More than 500	182	29.2
*Mother clinical stage during ANC*		
WHO stage 1	224	35.9
WHO stage 2	104	16.7
WHO stage 3	100	16
WHO stage 4	9	1.4
Undocumented	187	70
*PMTCT intervention in the mother*		
None/unknown	279	44.7
Yes	345	55.3
*Type of PMTCT intervention (mother)*		
AZT^b^ only	50	8
HAART^c^	295	47.3
None	105	16.8
Missing	174	27.9
*Infant PMTCT intervention*		
Sd NVP^d^	26	4.2
Sd NVP + AZT	279	44.7
NVP for 6 weeks	179	28.7
NVP >6 weeks	12	1.9
None	90	14.4
Missing	38	6.1
*Mother ANC*		
Yes	86	13.8
No	388	62.2
Missing	150	24

^a^Antenatal care

^b^Zidovudine

^c^Highly active antiretroviral therapy

^d^Nevirapine

Among the included infants, 27/624 (4.3 %) were positive for HIV-DNA PCR at the age of 6–8 weeks. Among 191 infants who took Nevirapine for 6 weeks or more, none of them had a positive HIV DNA PCR result. The highest positive result rate was observed for those who took either no prophylaxis or single dose Nevirapine (11.5 and 11.1 % respectively). And those who took combination of single dose Nevirapine and Zidovudine had a positivity rate of 3.9 %.

Various factors were identified as predictors of positivity of HIV DNA-PCR on the bivariate analysis. Being born at home (Crude OR, 3.26 95 % CI 1.24–8.53; p = 0.016), maternal CD4 count during pregnancy less than 100 cells/mm^3^ (Crude OR, 5.68 95 % CI 1.15–28.05; p = 0.033), absence of any PMTCT interventions for the mother (Crude OR, 6.10 95 % CI 2.45–15.17; p < 0.0001), absence of antenatal care follow up (Crude OR, 5.54 95 % CI 2.27–13.52; p < 0.0001), and mother not enrolled in HIV care and treatment during pregnancy (Crude OR, 4.32 95 % CI 1.66–11.24; p = 0.003) were associated with increased probability of HIV infection at 6–8 weeks. However, none of these variables were found to be independent predictors of positivity using multivariate logistic regression (Table 3).

**Table 3:** Associated factors with DNA PCR positivity at 6–8 weeks of age

Variable	DNA-PCR result		p value	Crude OR (95 % CI)	Adjusted OR (95 % CI)
	Positive (%)	Negative (%)			
*Sex*			0.992		
Male	14 (4.3)	309 (95.7)	0.992	1.00 (0.46–2.17)	
Female	13 (4.3)	288 (95.7)		Ref	
*Place of birth*			0.016		
Health facility	19 (3.6)	506 (96.4)		Ref	Ref
Home	6 (10.9)	49 (89.1)		3.26 (1.24–8.53)	3.10 (0.53–18.24)
*Type of feeding*			0.104		
Exclusive BF	16 (3.2)	478 (96.8)		Ref	
Exclusive RF	6 (6.5)	85 (93.5)		2.11 (0.80–5.54)	
Mixed feeding	3 (6.9)	28 (93.1)		0.66 (0.16–2.81)	
*Infant PMTCT*			0.000^a^	–	
Sd^b^ NVP or none	13 (11.2)	103 (88.8)			
Sd NVP + AZT	11 (3.9)	268 (96.1)			
Sd NVP ≥6 weeks	0 (0)	191 (100)			
*Mother CD4 during pregnancy*			0.033		
<100/mm^3^	3 (17.6)	14 (82.4)		5.68 (1.15–28.05)	4.50 (0.71–28.56)
100–500/mm^3^	3 (1.3)	236 (98.7)		0.34 (0.08–1.53)	0.26 (0.05–1.33)
>500/mm^3^	4 (3.6)	106 (96.4)		Ref	Ref
*Mother WHO stage*			0.344		
WHO stage 1	7 (3.3)	204 (96.7)		Ref	
WHO stage 2	2 (2.1)	91 (97.9)		0.64 (0.13–3.14)	
WHO stage 3/4	6 (6)	93 (94)		1.88 (0.62–5.75)	
*Mother any PMTCT*			0.000		
No	13 (12.7)	89 (87.3)		6.10 (2.45–15.17)	3.80 (0.68–21.09)
Yes	8 (2.3)	334 (97.7)		Ref	Ref
*Type of PMTCT (mother)*			0.402		
AZT only	2 (4)	48 (96)		2.01 (0.39–10.24)	
HAART	6 (2)	289 (98)		Ref	
*Antenatal care follow up*			0.000		
No	11 (12.8)	75 (87.2)		5.54 (2.27–13.52)	4.05 (0.25–65.75)
Yes	10 (2.6)	378 (97.4)		Ref	Ref
*Mother care and treatment*			0.003		
Enrolled in care and Rx^c^	14 (3.3)	406 (96.7)		Ref	–
Not enrolled	7 (13)	47 (87)		4.32 (1.66–11.24)	

^a^ *X* ^2^ test was used

^b^Single dose

^c^Treatment

## Discussion

Our findings showed that the infection rate at 6–8 weeks of DNA-PCR for HIV was at 4.3 %. This figure is small as compared to the study at St. Luke hospital, Gondar University Referral hospital, and Tanzania [17–19]. Infants who took PMTCT "Option B+" were all negative for HIV at 6–8 weeks while those who didn’t take any prophylaxis or those who took single dose Nevirapine had the highest infection rate. The findings underscore the importance of enhancing PMTCT interventions for both the mother and the infant at the earliest possible.

The current findings showed that there is a significantly high loss to follow up. Among all the enrolled infants, 199 (22.2 %) were lost to follow up. This figure is lower than 39.4 and 40.4 % lost to follow up rates by studies at St. Luke hospital and Mc Cord hospital of KwaZulu-Natal, South Africa respectively [18, 20]. The differences could be possibly from the time at measurement of loss to follow up, which in our case was at 6–8 weeks of age. Since the current study assessed loss to follow up at 6–8 weeks of age, it does not account for those infants who would be lost to follow up after the first DNA PCR and those who were excluded from the study. Hence, the current findings could be an underestimate of the overall problem of loss to follow up. The findings underscore for the need for better strategies to track and link HIV exposed infants to care and treatment throughout the time of exposure (during pregnancy, labor and delivery and breastfeeding).

One of the PMTCT interventions is starting mothers on prophylaxis as early as possible. In the current study, 123 (14.9 %) mothers during ANC and 95 (15.2 %) mothers during labor have not received any PMTCT intervention. Higher missed opportunities were reported by a group from St. Luke hospital; southwest Ethiopia in 2014 [18]. The differences could reflect the recent improvements in ANC coverage and provision of PMTCT interventions since our study addressed both the recent and old data. It can be taken as a clue for meeting the goal set by the Ethiopia FMOH to provide PMTCT prophylaxis for 85 % of eligible pregnant women by end of 2015 [21]. Findings from Johannesburg, South Africa showed a lower missed intra partum PMTCT intervention opportunity of 7.36 %; indicating the better health care in the setting [6]. Also, 90 (14.4 %) of the infants did not get any PMTCT intervention. This figure is lower when compared to a similar study done in southwest Ethiopia 163 (38.3 %) and in Gondar University hospital in 2013 both of which have similar HIV transmission dynamics to the current setting [18]; but slightly higher than Johannesburg study which was 12 (5.2 %) [6].

In the current study, possibly because of the relatively small number of babies who had a positive DNA PCR result and the higher ANC coverage, in contrast to the studies in St. Luke hospital and Gondar University Hospital which have a similar HIV transmission dynamics, ANC follow up, lack of PMTCT intervention and maternal WHO clinical stage were not associated with infant HIV infection at 6–8 weeks of age [17, 18].

In order to avoid the increased incidence of diarrhea, pneumonia, and death observed with replacement feeding, WHO recommends breastfeeding duration for 2 years for HIV exposed infants [22]. However, the risk of HIV infection among infants was found to be higher. Our findings showed that infants on breast feeding and complementary feeding were found to be three times more likely to have a DNA PCR result positive as compared to those on exclusive breast feeding. The difference in infection rate was reported to be higher in other similarly done studies [17, 18].

The limitations of the current study include its retrospective nature posing problems of incomplete recordings. Moreover, the data used in this study were not originally collected for research purposes and therefore the quality of the data might not be good. Since this is a record review, we cannot confidently confirm the accuracy of the information reported and documented by the clinicians. The transmission rate reported in the study is probably an underestimation of the actual MTCT since follow up data including transmission during breast feeding is not reported in the current study. The abstraction forms lacked information on year of enrolment and could not give more information on the number of infants enrolled in each specific year during the record review period.

## Conclusions

PMTCT ‘‘Option B+’’ resulted in zero infection rates among the included infants. There was a high loss to follow up rate even at 6–8 weeks of age. The authors recommend that a better strategy of linkage to care and treatment of HIV exposed infants to care and treatment should be devised.
